# Efficacy of 810 nm and 650 nm Diode Laser Alone and in Combination With Sodium Fluoride Gel in Treating Dentin Hypersensitivity: A Split-Mouth Randomized Clinical Study

**DOI:** 10.7759/cureus.33489

**Published:** 2023-01-07

**Authors:** Khalid Jomaa, Mahmoud Abdul-Hak, Wael H Almahdi, Mohammed Rasheed Al Namly, Louay Hanafi

**Affiliations:** 1 Department of Oral Medicine, Damascus University, Damascus, SYR; 2 Department of Periodontics, University of Damascus Faculty of Dentistry, Damascus, SYR; 3 Department of Fixed Prosthodontics, Damascus University, Damascus, SYR; 4 Department of Pediatric Dentistry, Damascus University, Damascus, SYR

**Keywords:** sodium fluoride (naf) gel, laser therapy, diode laser, dentine hypersensitivity, dentinal tubules

## Abstract

Introduction

Dentin hypersensitivity has been defined as a short, sharp pain caused by exposing naked dentin to external stimuli such as evaporation, heat, cold, osmotic liquids, and tactile effects. None of the available treatments has been considered the gold standard in treating dentin hypersensitivity. The use of laser has recently become more popular, and it is a promising treatment method in this field. The study aimed to compare the effectiveness of both 810 nm and 650 nm diode lasers in treating dentin hypersensitivity.

Materials and methods

This study was conducted on six patients who were referred to the Department of Oral Medicine, Faculty of Dental Medicine, Damascus University (4 females and 2 males) with 108 teeth suffering from generalized dentin hypersensitivity and were randomly divided into two groups. Group 1 consisted of three patients with 50 teeth treated with an 810 nm diode laser; it was divided by the split-mouth design into two subgroups. The first subgroup was treated by the application of the laser alone toward the target area with 1-watt power, continuous mode, and a total application time of 90 seconds, and the second subgroup was treated by applying sodium fluoride gel for one minute, then the laser was applied with the same settings as the first subgroup. Group 2 consisted of three patients with 58 teeth treated with a 650 nm diode laser; it was divided by the split-mouth design into two subgroups. The first subgroup was treated by the application of the laser alone toward the target area with 200 m watt power, continuous mode, and a total application time of 120 seconds, and the second subgroup was treated by applying sodium fluoride gel for one minute and then applying the laser with the same settings as the first subgroup. The pain was evaluated using a numeric rating scale (NRS) of 100 degrees before and immediately after treatment, and then after three to six and nine months by applying an air stream from a dental chair syringe at a distance of 0.5 cm from the target area.

Results

This study showed that both types of lasers were effective in treating pain caused by dentinal hypersensitivity. The average values ​​of pain reduction on the NRS showed the superiority of the 810 nm diode laser over the 650 nm after treatment and at all time points of pain assessment. No statistically significant differences were detected between applying laser alone and applying it combined with sodium fluoride gel in pain reduction values.

Conclusions

The application of an 810 nm diode laser either alone or in combination with sodium fluoride gel in treating dentin hypersensitivity is effective and better than the application of a 650 nm diode laser either alone or in combination with sodium fluoride gel. In addition, applying a 650 nm diode laser either alone or in combination with sodium fluoride gel has slight effectiveness in treating dentine hypersensitivity, and it is believed that a single treatment session with a 650 nm diode laser was not enough to obtain the required pain reduction.

## Introduction

Dentinal hypersensitivity (DH) is defined as a sharp, short pain that appears after exposing the exposed dentin to external stimuli, e.g., evaporative, thermal, lytic, or tactile stimuli. This pain cannot be attributed to other dental lesions or diseases [1]. DH causes real daily suffering for many people, and the mechanism of the occurrence is not completely clear. It is described as a multifactorial case of multiple etiologies and risk factors, e.g., tooth wear and gingival recession [2].

The number of people with dentin hypersensitivity has increased in recent decades. A systematic review by Zeola and his colleagues of 77 worldwide studies about the prevalence of DH in 2019 has revealed that the mean prevalence of DH among the reviewed studies was 33.5% [3].

The pain of DH has been explained based on many theories. The hydrodynamic theory is considered one of the most acceptable theories for most researchers in this field. Its interpretation of pain depends on the movement of fluids inside dentinal tubules, either inwards or outwards of the pulp. This theory was presented by Brannström [4]. It assumes that the external stimuli applied to the exposed dentine lead to disturbance and an increase in the movement and flow of fluids within the dentinal tubules, which leads to the activation of the nerve located in the pulpal endings of the dentinal tubules [5].

The reasons for DH are the following: First, the exposed dentine, which is called localization. The second reason is the opening of the dentinal tubules directly connected to the pulp, called initiation [2]. The exposed dentine results either from gingival recession due to gingivitis, periodontitis, wrong tooth brushing methods, or periodontal surgeries [6] or from enamel loss caused by erosion, attrition, and abrasion [7].

DH can be controlled by either an at-home treatment modality, as it depends on the patient such as using toothpaste containing fluoride, strontium, and oxalate, or by an in-office treatment modality under the supervision of the dentist who has a wide range of products that can be applied to treat DH such as varnishes, resin-based materials and lasers [2]. In many cases, it is necessary to apply both methods, as when the efforts of both the dentist and the patient are combined to reach an integrated treatment plan [2,8].

In 1935, scientist Grossman suggested several conditions that must be met in the ideal treatment of dentin hypersensitivity [9]. Although these conditions have been discussed for a long time, they are still good and acceptable. The ideal treatment for DH should be easy to apply, quick-acting, long-term efficacy, continuous effect, non-irritating to the dental pulp, non-painful during the application, and not cause tooth staining. To date, no treatment method has all of these qualities [10].

With the insertion of medical laser devices in various fields of dentistry, the first laser was used in the mid-sixties to remove dental necrosis. However, the first time laser was applied in the treatment of DH was in the mid-eighties by Matsumoto and his colleagues [11].

Compared to other methods of treating DH, the use of laser is an easy-to-apply, safe and reliable method, in addition to having an immediate effect [12,13].

The recent great development of the laser industry, especially in multi-wavelength diode lasers, and their widespread availability make them the best choice for most dentists. These devices are characterized by their small size and ease of use, movement, and operation through modern interactive interfaces. Several systematic reviews have concluded that diode and near-infrared lasers have been effective in treating DH [14,15].

After reviewing the medical literature, systematic reviews suggested that it is necessary to design more randomized controlled trials with long follow-up periods to ensure the therapeutic effect of the laser on DH [16,17]. it was also found that there was only one study comparing the effectiveness of both 660 nm and 810 nm diode lasers in treating DH, and proved that both lasers were effective in this field, and the 810 nm diode laser was superior and more long-lasting than the 660 nm diode laser [10]. No study discussed the effectiveness of the 650 nm diode in combination with sodium fluoride gel in treating dentin hypersensitivity. Therefore, the aim of this nine-month randomized clinical study with a split-mouth design was to compare the effectiveness of a 650 nm diode laser and an 810 nm diode laser, either alone, or in combination with sodium fluoride gel in treating DH.

## Materials and methods

Study sample

This was a single-blind, randomized clinical trial with a split-mouth design. This study was conducted after getting approval from the Scientific Research Ethics Committee at the Faculty of Dentistry, Damascus University (UDDS-834-03092019/SRC-1450), and was funded by the Damascus University Postgraduate Research Budget. The sample included six patients (four females and two males) suffering from generalized DH; their ages ranged between 20 and 30 years. The sample was collected from patients visiting the Department of Oral Medicine at the Faculty of Dentistry, Damascus University, Syrian Arab Republic from 6/1/2020 to 12/31/2021. Patients were accepted after they were provided with all information related to the research (the method of conducting this study and the follow-up period) in a written form, and all of their questions were answered orally. After that, written consent to enter this research was taken.

Study groups

The sample size was determined using the G power program (Heinrich-Heine-Universität, Düsseldorf, Germany), and depending on the mean and standard deviation in the study of Naghsh and his colleagues [10]. After adopting the study power to 85% and its significance to 0.05, it was found that the number of teeth in each group must be 52.

Patients included in the study were divided randomly into two main groups by drawing a card with a hidden number from a box without knowing the meaning of the number that appeared.

Inclusion criteria

Patients were selected according to the following inclusion criteria: patient age range 18 to 30 years, with good general health and did not suffer from any general or chronic diseases, presenting with at least one tooth with DH on each side of the upper or lower jaw. The pain intensity of the tooth with DH was 30 or higher on the NRS numeric rating scale, and the area of ​​DH was the vestibular or lingual surfaces of the incisors, canines, and upper and lower premolars.

Exclusion criteria

Patients were excluded according to the following criteria: patients undergoing periodontal treatment one month prior to the study, periodontal surgery three months prior to the study, and fixed orthodontic treatment. Exclusion criteria also comprised patients consuming anti-inflammatory drugs, analgesics, and sedatives 72 hours before laser application. After examining the patient, molars and all teeth that were decayed, broken, cracked, having birth defects in enamel or dentin, restoratively treated, crowned, used as abutments, or showing symptoms of irreversible pulpitis, were excluded from the studied sample. Ethyl chloride was used to test the vitality of the teeth and to exclude teeth showing symptoms of irreversible pulpitis.

Group 1: 810 nm Diode Laser Group

The number of teeth in this group was 60, and after applying the inclusion and exclusion criteria, the number decreased to 50 teeth treated with an 810 nm diode laser, (Pioon Multi-wavelength Laser, Wuhan, China), 1-watt power, continuous mode, and a total application time of 90 seconds.

By using the split-mouth design, this group was divided into two subgroups.

The first subgroup was the right-handed split of the upper/lower jaw and included 25 teeth suffering from DH, which were treated using an 810 nm diode laser alone. The second subgroup was the left-handed split of the upper/lower jaw and included 25 teeth suffering from DH, which were treated by applying sodium fluoride gel to the area of DH. Then, the laser was applied with the same settings as the first subgroup.

Group 2: 650 nm Diode Laser Group

The number of teeth in this group was also 60, and after applying the inclusion and exclusion criteria, the number decreased to 58 teeth treated with a 650 nm diode laser (Pioon Multi-wavelength Laser, Wuhan, China), 200 m watt power, continuous mode, and a total application time of 120 seconds.

By using the split-mouth design, this group was divided into two subgroups.

The first subgroup was the right-handed split of the upper/lower jaw and included 29 teeth suffering from DH, which were treated by applying a 650 nm diode laser beam alone. The second subgroup was the left-handed split of the upper/lower jaw and included 29 teeth suffering from DH, which were treated by applying sodium fluoride gel to the area of ​​DH. Then, the laser was applied with the same settings as the first subgroup.

Pain assessment

**Figure 1:**
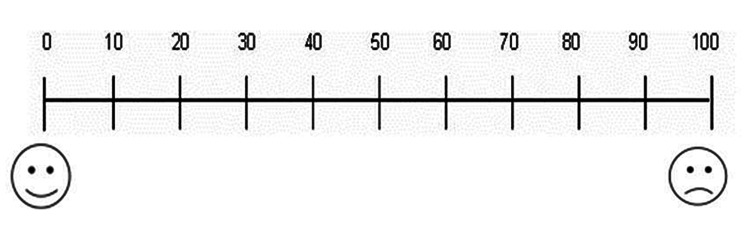
Numeric rating scale

The numeric rating scale NRS was used to assess pain. It consists of 100 degrees. The method of measuring pain was explained to each patient. Each patient was asked to respond verbally about the value of the pain she/he felt. The response was determined following the scale displayed in front of the patient, in which the number 0 indicates no pain at all, and the number 100 indicates very severe and unbearable pain, mild pain ranges from 1 to 30, moderate pain is greater than 30 to 60, and severe pain is greater than 60 (Figure 1).

**Figure 2:**
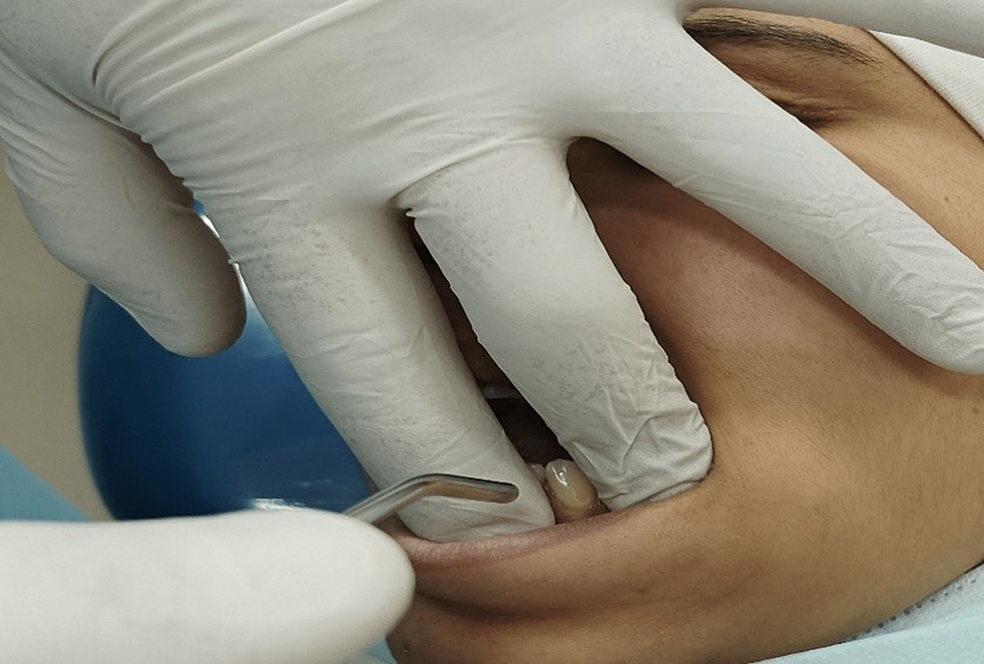
The method of isolating the target tooth with the application of the evaporative stimulus

The pain was assessed for each tooth included in the study separately, as the researcher isolated the target tooth by putting his fingers on the adjacent teeth (Figure 2).

After examining all the teeth of each patient, teeth that did not meet the study criteria were excluded from the study. Then, dry induction (vaporization) was used to assess the degree of pain in teeth in addition to dentin hypersensitivity. This was done by applying a continuous air stream for three seconds from the air syringe in the dental chair, directly on the tooth's surface that suffers from DH and a distance of about 0.5 cm. The temperature of the air stream was similar to the room temperature and the average was 25°C at all time points of pain assessment, and this temperature was stable all the time and controlled by the room AC, which was set up at the same degree (25°).

Patients were also emphasized that it was necessary to follow oral health procedures by brushing their teeth daily (at least twice) with a medium-hard toothbrush without using any special pastes to treat DH and avoiding whitening toothpaste during treatment follow-up periods.

Clinical procedures

All clinical procedures were done by one researcher, and only the patient was blinded.

Teeth were isolated using a cotton roll in the vestibular gutter, and the DH area was dried with a small cotton ball. An air stream from the air syringe of the dental chair was applied perpendicular to the vestibular surface for three seconds. The pain value given by the patient was recorded immediately prior to treatment.

After that, the laser was applied in each of the two study groups according to the following:

810 nm Diode Laser Group

**Figure 3:**
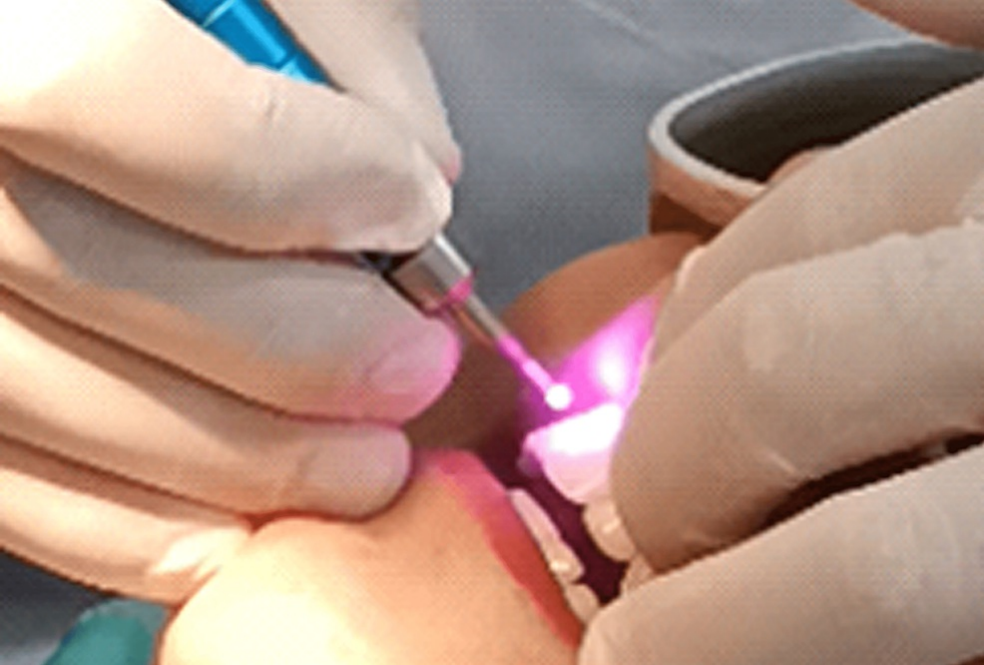
Application method of the 810 nm diode laser

The first subgroup was treated by applying a diode laser beam of 810 nm in a continuous mode with a power of 1 watt for 30 seconds. The application was perpendicular to the tooth's surface and at a distance of 2 mm by means of an optical fiber with a diameter of 320 micrometers. Thus, the entire area of DH was slowly scanned. The application was repeated three times with an interval of 30 seconds between each application. The total application time was 90 seconds. The second subgroup was treated by applying sodium fluoride gel to the area of ​​DH with a small brush and left for one minute. Then, the extra gel was removed with a small cotton ball. After that, the laser was applied with the same settings used in the first category (Figure 3).

650 nm Diode Laser Group

**Figure 4:**
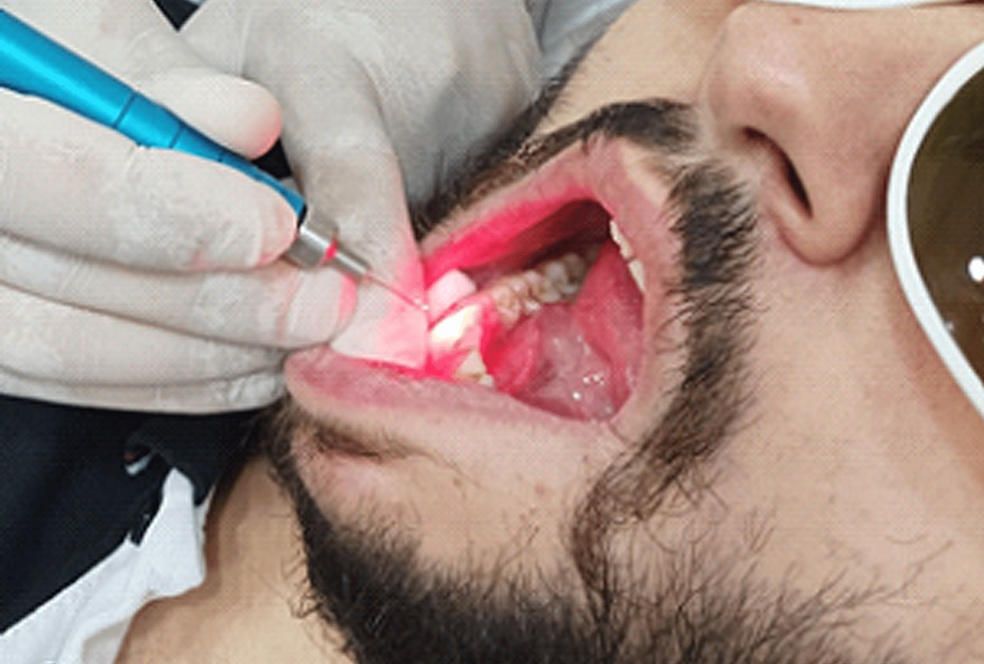
Application method of the 650 nm diode laser

The first subgroup was treated by applying a diode laser beam of 650 nm in a continuous system with a power of 200m watts for 30 seconds. The application was perpendicular to the tooth's surface and at a distance of 2 mm by means of an optical fiber with a diameter of 320 micrometers. Thus, the entire area with DH was slowly scanned. The application was repeated four times with an interval of 30 seconds between each application, and the total application time was 120 seconds. The second subgroup was treated by applying sodium fluoride gel to the area of ​​DH with a small brush and left for one minute. Then, the extra gel was removed with a small cotton ball. After that, the laser was applied with the same previous settings (Figure 4).

Afterward, the pain was re-assessed immediately after the treatment by applying an evaporation stimulus (air stream from the dental chair syringe to the vestibular region of the tooth for three seconds) at a room temperature of 25°C, and the pain value given by the patient was recorded.

Follow-up periods: The pain was re-assessed after three months, six months, and nine months.

Statistical study

Data were collected and entered into a Microsoft Excel sheet (Microsoft Corporation, Redmond, WA). They were analyzed statistically using SPSS® version 26.0 (IBM Corp., Redmond, NY), with a significance level of 0.05.

Independent samples' student t-tests were used to study the effects of the type of laser used and sodium fluoride gel application on the amount of pain reduction according to the NRS.

## Results

The study sample included six patients (4 females and 2 males) with 108 teeth suffering from dentin hypersensitivity according to the 100-degree numeric rating scale. There were fifty teeth in three patients (two females and one male) in the 810 nm diode laser group; teeth were 23 upper (5 premolars and 18 incisors) and 27 lower (9 premolars and 18 incisors). 

In 58 teeth in three patients (two females and one male) in the 650 nm diode laser group, teeth were 30 upper (11 premolars and 19 incisors) and 28 lower (9 premolars and 19 incisors) (Table 1).

**Table 1:** The effect of the type of laser on the amount of pain reduction according to NRS V: arithmetic mean ± standard deviation of the amount of pain reduction, Ϯ: the application of T-test for independent samples, *: statistically significant at P≤0.05, NRS: numeric rating scale

Time	Applying NaF	V laser 650nm (I)	V laser 810nm (J)	Mean Difference (I - J)	P value Ϯ	Significance.
Immediately post-treatment	No	7.2 ± 3.1	25.2 ± 6	-18	0.00	*
	Yes	7.5 ± 3.5	29.8 ± 4.7	-22.3	0.00	*
After 3 Months	No	7.8 ± 3.1	26.8 ± 7.1	-19	0.00	*
	Yes	8 ± 3.4	30.6 ± 5.3	-22.6	0.00	*
After 6 Months	No	8.8 ± 3.4	27 ± 6.9	-18.2	0.00	*
	Yes	8.9 ± 3.4	30.4 ± 5.8	-21.5	0.00	*
After 9 Months	No	9 ± 2.8	26.8 ± 6.4	-17.8	0.00	*
	Yes	9.6 ± 3.8	30.4 ± 4.8	-20.8	0.00	*

When applying a comparison of the effectiveness of the two types of lasers according to the pain reduction scores on the NRS, the results presented in Table 1 indicate that the 810 nm diode laser was superior over the 650 nm diode laser by a statistically significant difference (P ≤ 0.000). This superiority was noticed by applying the laser alone or by combining the laser with sodium fluoride immediately after treatment and in all follow-up periods. The mean pain reduction after nine months in the 810 diode laser alone group was 6.4 ± 26.8, while in the combination group, the mean pain reduction was 4.8 ± 30.4. On the other hand, the average pain reductions in the group of 650 diode laser alone and in combination with sodium fluoride gel were 2.8 ± 9 and 3.8 ± 9.6, respectively (Table 2).

**Table 2:** The effect of laser application alone or in combination with sodium fluoride gel on the amount of pain reduction according to NRS V: arithmetic mean ± standard deviation of the amount of pain reduction, Ϯ: the application of T-test for independent samples, *: statistically significant at P≤0.05, NRS: numeric rating scale

Group	Time	V without NaF (I)	V with NaF (J)	Mean Difference (I – J)	P value Ϯ	Significance.
Laser 650 nm	Immediately post-treatment	7.2 ± 3.1	7.5 ± 3.5	-0.3	0.702	NS
	After 3 Months	7.8 ± 3.1	8 ± 3.4	-0.2	0.815	NS
	After 6 Months	8.8 ± 3.4	8.9 ± 3.4	-0.1	0.916	NS
	After 9 Months	9 ± 2.8	9.6 ± 3.8	-0.6	0.464	NS
Laser 810 nm	Immediately post-treatment	25.2 ± 6	29.8 ± 4.7	-4.6	0.004	*
	After 3 Months	26.8 ± 7.1	30.6 ± 5.3	-3.8	0.036	*
	After 6 Months	27 ± 6.9	30.4 ± 5.8	-3.4	0.065	NS
	After 9 Months	26.8 ± 6.4	30.4 ± 4.8	-3.6	0.029	*

In the 650 nm diode laser group, there were no statistically significant differences between the application of the laser alone and the combined application with sodium fluoride gel after immediate treatment and during all follow-up periods. However, it can be clearly noticed that the combined application contributed to reducing the pain more than the laser alone (Table 2).

As for the 810 nm diode laser group, the results showed that the combined application of the laser with sodium fluoride gel was superior to the application of the laser alone in pain reduction. The difference was statistically significant immediately after treatment and during the follow-up periods, except after six months, as the difference was not statistically significant (P = 0.065). The average pain reduction was 6.9 ± 27 and 5.8 ± 30.4 when applying the laser alone and after sodium fluoride gel, respectively.

## Discussion

Dentin hypersensitivity is a serious challenge for many people and significantly affects their quality of life. It often forces them to change their daily habits such as eating, drinking, talking, and even brushing their teeth. Severe degrees of DH that last long periods may lead to psychological and emotional harm [18].

The global prevalence of DH ranges between 3% and 98%. This wide range is due to the different areas studied and the inclusion and diagnostic criteria applied in each study. In addition, it is ascribed to the different health and nutritional habits prevalent in a region [7]. The number of people who have visited dental clinics to obtain effective treatment for DH at a reasonable cost has increased recently. Therefore, this study was conducted to test the effectiveness of modern, widespread, low-priced laser devices in treating DH.

The participants' ages in this study ranged between 20 - 30 years. The split-mouth design was used, and the evaporative stimulator was used as an external stimulus for pain assessment. This protocol is consistent with the recommendations of the systematic review by Marto and his colleagues [19], as this review recommended the need to homogenize the ages of the groups. That is due to the different causes predisposing to have DH according to age. It also recommended using the split-mouth technique as possible since it enables researchers to test different treatment techniques for the same patient, and it indicates that the evaporative stimulus is considered the most accurate external stimulus in pain assessment. The same systematic review recommended using a mechanical stimulus by means of a dental probe in combination with the evaporative stimulus to obtain more accurate results. However, the current study did not use a mechanical stimulus, e.g., a dental probe due to the fear of the possibility of causing scratches on the surface of the tooth, which could lead to developing subsequent problems, and the area of ​​DH may be smaller than the diameter of the tip of the probe used. Thus, the mechanical stimuli could give a false result [20].

According to many previous studies, the current study adopted the teeth as the main research unit in the sample [10,21], in contrast to many other studies that adopted the person as the main research unit. Those who have relied on people justify their choice by the fact that the feeling of pain differs from one person to another. Therefore, it is necessary to conduct tests on the largest possible number of people for the results to be more objective and accurate [22].

The current study aimed to evaluate the effectiveness of the two lasers in reducing DH pain. The results showed that both types of laser contributed to reducing pain immediately after treatment, and the decrease continued during all observation periods. According to the literature, the immediate effect of both lasers can be attributed to their direct effect on the non-myelinated fibers (C) within the dental pulp. The laser depolarizes the nerve endings of these fibers and raises the pain threshold by activating the sodium-potassium pump within the cell membrane of these nerve endings [23]. Some studies indicated that the immediate effect of the 810 nm diode laser results from its ability to reduce the diameters of the dentinal tubules [24]. As for the long-term effect of both lasers, it can be attributed, according to the literature, to the ability of low-energy lasers to activate metabolic processes within odontoblasts and stimulate them to produce tertiary dentin [25]. This dentin contributes to the closure of the dentin tubules toward the direction of the dental pulp and thus leads to more isolation of the nerve fibers from external stimuli. In addition, these wavelengths stimulate blood circulation in the area and have an analgesic and anti-inflammatory effect [26].

The results of the current study showed that the application of the 810 nm diode laser according to the following settings (1 watt, continuous pattern, 90 seconds total application time) contributed effectively to the reduction of pain caused by DH immediately after treatment. The reduction continued during the follow-up periods, which extended to nine months. These results are consistent with the study by Hashim and his colleagues [21], in which the Diode 810 nm laser was applied according to the following settings (1 watt, continuous pattern, for 30 seconds and 60 seconds). However, the follow-up period lasted only two weeks, and the results of the current study are consistent with the results of the study conducted by Yilmaz and his colleagues [22]. They showed that the application of an 810 nm diode laser with an output power of 0.5 watts and an application time of 60 seconds with a follow-up period of six months was effective in treating DH. The two previous studies recommended that the application of lasers to treat DH is an easy, painless, and effective method, which is confirmed by the current study.

As for the 650 nm diode laser, the results of the current study showed that it reduced pain levels immediately after treatment, and the reduction continued during follow-up periods of up to nine months. However, this decrease was slight, as it did not exceed 10 degrees on the NRS of 100 degrees. On the other hand, in the study conducted by Dilsiz and his colleagues, in which a 685 nm diode laser was used with an output power of 25 mW and a frequency of 10 Hz for 100 seconds; the results showed that the previous laser was effective in reducing pain by 4 degrees of the 10-point visual analog (VAS) pain scale [27]. This difference could be attributed to the fact that the 650 nm diode laser application protocol in the current study consisted of only one treatment session while the 685 nm diode laser application protocol in the study conducted by Dilsiz and his colleagues consisted of three treatment sessions separated by 14 days each [27]. The current study's results differed from those of the study conducted by Naghsh and his colleagues, in which a 660 nm diode laser was used with an output power of 30 mW for 120 seconds. Their study showed a reduction of 5 degrees in the VAS pain scale after the fourth treatment session [10]. Both studies showed the superiority of the 810 nm diode laser over the 660 nm diode laser in Naghsh's study and the 650 nm diode laser in the current study.

The current study showed the superiority of the combined application of both types of lasers with sodium fluoride gel over the application of laser alone. However, there were no statistically significant differences in the 650 nm diode laser group. Nonetheless, by observing the pain reduction averages and the differences between them in Table 2, it can be said that combining both types of laser with sodium fluoride was more effective in reducing the pain of DH than the application of both types of lasers alone. This result can be explained by the synergy of the laser effect with the effect of fluoride ions in closing the dentinal tubules. These results are largely consistent with the study conducted by Gojkov-Vukelic and his colleagues [28].

Study limitations

In this study, teeth were considered the main unit in the studied sample, and since pain is a personal criterion that differs from one person to another, it was considered one of the most critical limitations of this study. In addition, there was no control group to control the results and exclude the placebo effect. That leads to proposing designs for future studies that consider persons - not teeth - as the main unit in the research sample, with a negative control group to control the results.

## Conclusions

Based on the results of this study, it can be concluded that the effectiveness of applying the 810 nm diode laser alone and in combination with sodium fluoride gel to treat dentine hypersensitivity was better than the application of the 650 nm diode laser alone and in combination with sodium fluoride gel. Moreover, the combined technique resulted in higher pain reduction than the application of the 810 nm diode laser alone.

On the other hand, the application of the 650 nm diode laser alone and in combination with sodium fluoride gel had slight effectiveness in treating dentine hypersensitivity. In addition, the combined technique resulted in higher pain reduction than the application of this laser alone, even though there were no statistically significant differences between the two application methods, and it is believed that the single treatment session with the 650nm diode laser was not enough to obtain the required pain reduction.

## References

[1] Mahdian M, Behboodi S, Ogata Y, Natto ZS (2021). Laser therapy for dentinal hypersensitivity. Cochrane Database Syst Rev.

[2] West N, Seong J, Davies M (2014). Dentine hypersensitivity. Monogr Oral Sci.

[3] Favaro Zeola L, Soares PV, Cunha-Cruz J (2019). Prevalence of dentin hypersensitivity: systematic review and meta-analysis. J Dent.

[4] Brannstrom M (1966). Sensitivity of dentine. Oral Surg Oral Med Oral Pathol.

[5] Longridge NN, Youngson CC (2019). Dental pain: dentine sensitivity, hypersensitivity and cracked tooth syndrome. Prim Dent J.

[6] West NX, Lussi A, Seong J (2013). Dentin hypersensitivity: pain mechanisms and aetiology of exposed cervical dentin. Clin Oral Investig.

[7] Alcântara PM, Barroso NF, Botelho AM, Douglas-de-Oliveira DW, Gonçalves PF, Flecha OD (2018). Associated factors to cervical dentin hypersensitivity in adults: a transversal study. BMC Oral Health.

[8] Orchardson R, Gillam DG (2006). Managing dentin hypersensitivity. J Am Dent Assoc.

[9] Kimura Y, Wilder-Smith P, Yonaga K, Matsumoto K (2000). Treatment of dentine hypersensitivity by lasers: a review. J Clin Periodontol.

[10] Naghsh N, Kachuie M, Kachuie M, Birang R (2020). Evaluation of the effects of 660-nm and 810-nm low-level diode lasers on the treatment of dentin hypersensitivity. J Lasers Med Sci.

[11] Varma SR, AlShayeb M, Narayanan J, Abuhijleh E, Hadi A, Jaber M, Abu Fanas S (2020). Applications of lasers in refractory periodontitis: a narrative review. J Int Soc Prev Community Dent.

[12] Hu ML, Zheng G, Han JM, Yang M, Zhang YD, Lin H (2019). Effect of lasers on dentine hypersensitivity: evidence from a meta-analysis. J Evid Based Dent Pract.

[13] Simões TM, Melo KC, Fernandes-Neto JA (2021). Use of high- and low-intensity lasers in the treatment of dentin hypersensitivity: a literature review. J Clin Exp Dent.

[14] Abdelkarim-Elafifi H, Parada-Avendaño I, Arnabat-Domínguez J (2022). Parameters used with diode lasers (808-980 nm) in dentin hypersensitivity management: a systematic review. J Lasers Med Sci.

[15] Bellal S, Feghali RE, Mehta A, Namachivayam A, Benedicenti S (2022). Efficacy of near infrared dental lasers on dentinal hypersensitivity: a meta-analysis of randomized controlled clinical trials. Lasers Med Sci.

[16] Shan Z, Ji J, McGrath C, Gu M, Yang Y (2021). Effects of low-level light therapy on dentin hypersensitivity: a systematic review and meta-analysis. Clin Oral Investig.

[17] Machado AC, Viana ÍEL, Farias-Neto AM, Braga MM, de Paula Eduardo C, de Freitas PM, Aranha AC (2018). Is photobiomodulation (PBM) effective for the treatment of dentin hypersensitivity? A systematic review. Lasers Med Sci.

[18] Liu XX, Tenenbaum HC, Wilder RS, Quock R, Hewlett ER, Ren YF (2020). Pathogenesis, diagnosis and management of dentin hypersensitivity: an evidence-based overview for dental practitioners. BMC Oral Health.

[19] Marto CM, Baptista Paula A, Nunes T (2019). Evaluation of the efficacy of dentin hypersensitivity treatments-a systematic review and follow-up analysis. J Oral Rehabil.

[20] Ricarte JM, Matoses VF, Faus Llácer VJ, Flichy Fernández AJ, Mateos Moreno B (2008). Dentinal sensitivity: concept and methodology for its objective evaluation. Med Oral Patol Oral Cir Bucal.

[21] Hashim NT, Gasmalla BG, Sabahelkheir AH, Awooda AM (2014). Effect of the clinical application of the diode laser (810 nm) in the treatment of dentine hypersensitivity. BMC Res Notes.

[22] Yilmaz HG, Kurtulmus-Yilmaz S, Cengiz E (2011). Long-term effect of diode laser irradiation compared to sodium fluoride varnish in the treatment of dentine hypersensitivity in periodontal maintenance patients: a randomized controlled clinical study. Photomed Laser Surg.

[23] Bagis S, Comelekoglu U, Sahin G, Buyukakilli B, Erdogan C, Kanik A (2002). Acute electrophysiologic effect of pulsed gallium-arsenide low energy laser irradiation on configuration of compound nerve action potential and nerve excitability. Lasers Surg Med.

[24] Umana M, Heysselaer D, Tielemans M, Compere P, Zeinoun T, Nammour S (2013). Dentinal tubules sealing by means of diode lasers (810 and 980 nm): a preliminary in vitro study. Photomed Laser Surg.

[25] Dilsiz A, Aydın T, Emrem G (2010). Effects of the combined desensitizing dentifrice and diode laser therapy in the treatment of desensitization of teeth with gingival recession. Photomed Laser Surg.

[26] Yilmaz HG, Kusakci-Seker B, Bayindir H, Tözüm TF (2010). Low-level laser therapy in the treatment of mucous membrane pemphigoid: a promising procedure. J Periodontol.

[27] Dilsiz A, Canakci V, Ozdemir A, Kaya Y (2009). Clinical evaluation of Nd:YAG and 685-nm diode laser therapy for desensitization of teeth with gingival recession. Photomed Laser Surg.

[28] Gojkov-Vukelic M, Hadzic S, Jahic IM, Pasic E, Muharemovic A (2021). Comparative evaluation of the effects of diode laser and desensitizing agents on the treatment of dentin hypersensitivity: a clinical study. Acta Inform Med.

